# Prevalence of glucose-6-phosphate dehydrogenase (G6PD) deficiency among the fulminant hepatitis A virus infection patients

**DOI:** 10.22088/cjim.15.3.451

**Published:** 2024-08-01

**Authors:** Fardad Ejtehadi, Maryam Sadat Serpoosh, Iraj Shahramian, Ladan Aminlari, Ramin Niknam, Gholam Reza Sivandzadeh, Masoud Tahani, Amin Javadifar, Fateme Sharafi, Maryam Moini

**Affiliations:** 1 Gastroenterohepatology Research Center, Shiraz University of Medical Sciences, Shiraz, Iran; 2 Professor, Department of Pediatrics, School of Medicine, Shiraz University of Medical Sciences, Shiraz, Iran; 3 Associated Professor of Gastroenterology and Hepatology, Department of Internal Medicine, Gastroenterohepatology Research Center, School of Medicine, Shiraz University of Medical Sciences, Shiraz, Iran; 4 Pediatric Gastroenterology and Hepatology Research Center, Zabol University of Medical Sciences, Zabol, Iran; 5 Immunology Research Center, Inflammation and Inflammatory Diseases Division, Mashhad University of Medical Sciences, Mashhad, Iran; 6 Division of Gastroenterology, University of Ottawa, Ottawa, Canada

**Keywords:** G6PD Deficiency, Hepatitis A, Fulminant hepatitis

## Abstract

**Background:**

Hepatitis A is a widespread viral infection with significant public health implications. Assessing glucose 6-phosphate dehydrogenase (G6PD) deficiency in hepatitis A patients is essential for various reasons, including prognosis, disease severity evaluation, encephalopathy risk identification, tailored management, and advancing scientific understanding. This study aimed to investigate the prevalence and clinical implications of G6PD impairment in individuals with fulminant hepatitis A.

**Methods:**

A cross-sectional descriptive analysis was conducted, involving hospitalized patients with fulminant hepatitis A. Demographic data, prevalence rates, and clinical findings were recorded in a database. The diagnosis of hepatitis A infection was confirmed using an anti-HAV IgM antibody test, and G6PD enzyme activity was measured with a fluorescent spot assay.

**Results:**

Out of 81 patients with hepatitis A, 57 (70.4%) were males, and 24 (29.5%) were females, with an average age of 24.6 years. Dark yellow urine and anorexia were the most common clinical symptoms. Notably, 30 (37%) patients lacked G6PD. The group with G6PD deficiency showed significantly higher rates of encephalopathy and mortality (P<0.01), along with elevated bilirubin (P=0.00), abnormal coagulation parameters, and low hemoglobin levels (P=0.00).

**Conclusion:**

In light of these findings, the present study proposes the implementation of routine G6PD level assessments and the evaluation of other relevant markers in regions where hepatitis A is endemic. Furthermore, the study underscores the need for vigilant monitoring of hemolysis and encephalopathy in affected patients to optimize clinical management and reduce morbidity and mortality associated with this condition.

Viral hepatitis is a systemic infection that primarily targets the liver. It is caused by a group of microorganisms, including the hepatitis A virus (HAV), hepatitis E virus (HEV), hepatitis B virus (HBV), hepatitis C virus (HCV), and hepatitis D virus (HDV). While HAV and HEV are transmitted through contaminated food and water, HBV, HCV, and HDV are mainly transmitted through blood or sexual contact (1). Among these viruses, HAV infection is particularly common in children and is a leading cause of acute hepatitis and liver failure in this age group. 

Crowded conditions with poor hygiene contribute to the increased prevalence of HAV infection, which is primarily transmitted through person-to-person contact. However, rare cases of HAV transmission through contaminated blood products or needles during blood transfusions have also been reported. Vertical transmission from mother to infant is another documented route of infection (2, 3).

Fulminant hepatitis is a severe form of liver damage, often leading to hepatic encephalopathy within eight weeks of jaundice onset. In the subfulminant form, encephalopathy occurs after eight weeks but within six months of symptom onset (5). Complications associated with hepatitis A include bacterial or fungal infections, kidney failure, lung failure, and electrolyte imbalances, all of which can worsen the prognosis (6). While less than 1% of acute HAV infections progress to acute liver failure (ALF), adults, especially those with pre-existing liver damage like non-alcoholic fatty liver disease (NAFLD) or alcoholic steatohepatitis (ASH), are at a higher risk of developing acute-on-chronic liver failure (7, 8).

 Fulminant hepatitis A virus (HAV) infection is a rare but potentially life-threatening condition characterized by rapid liver failure and a high mortality rate. While the majority of individuals infected with HAV experience a self-limiting illness, a small subset progresses to fulminant hepatitis A, which requires intensive care and, in severe cases, liver transplantation (9, 10). Fulminant hepatitis A typically manifests with a sudden onset of severe symptoms and rapidly progresses to liver failure within a short period, usually within a few days to weeks. Common clinical features include jaundice, hepatomegaly, coagulopathy, hepatic encephalopathy (brain dysfunction due to liver failure), and multi-organ failure. Due to the rapid deterioration of liver function, individuals with fulminant hepatitis A require prompt medical attention and specialized care, including close monitoring in intensive care units (5, 11). 

The precise mechanisms underlying the development of fulminant hepatitis A are not fully understood. It is believed that certain host and viral factors contribute to the progression of the disease. Host factors include age, sex, immunological status, and genetic predisposition, while viral factors include the viral load, virulence, and specific HAV strains. However, the interplay between these factors and the exact pathogenesis of fulminant hepatitis A are still areas of ongoing research (10, 11).

 G6PD deficiency is an X-linked enzymatic disorder affecting the red blood cells, leading to impaired redox balance and increased susceptibility to oxidative stress. The condition is prevalent in various populations, particularly in regions where malaria is or has been endemic due to the conferred protection against malaria parasites. While the association between G6PD deficiency and various infectious diseases has been studied extensively, its relationship with fulminant HAV infection remains relatively unexplored (12, 13, 14).

 Patients with G6PD deficiency and hepatitis A displayed distinct laboratory findings indicative of more severe disease. Understanding the prevalence of G6PD deficiency among patients with fulminant HAV infection is crucial for identifying potential risk factors and unraveling the underlying pathophysiological mechanisms that may contribute to disease severity. Moreover, such knowledge may have important implications for clinical management strategies, including the identification of high-risk individuals and the development of tailored therapeutic interventions (15-17). 

Therefore, by analyzing available data on disease severity, mortality rates, and complications, we aim to identify any significant associations between G6PD deficiency and adverse clinical outcomes in patients with fulminant HAV infection. These findings may have important implications for risk stratification, prognostication, and therapeutic decision-making.

## Methods

The present study was a cross-sectional descriptive study conducted at Namazi Hospital, Shiraz University of Medical Sciences between September 2012 and September 2018. 

**Inclusion Criteria:** The inclusion criteria for the study were as follows:

1. Age: All patients over 18 years of age diagnosed with fulminant hepatitis A virus during the study period were included. The minimum age requirement was set to ensure the inclusion of adult patients.

2. Diagnosis of Fulminant Hepatitis A: Fulminant hepatitis A was defined as a severe form of acute hepatitis A characterized by rapid liver failure and associated complications. Clinical criteria for fulminant hepatitis A included the sudden onset of severe jaundice, coagulopathy, hepatic encephalopathy, and rapid progression to liver failure within a short period.

3. Laboratory Criteria: Patients needed to meet specific laboratory criteria for the diagnosis of fulminant hepatitis A. This included markedly elevated serum transaminases (AST and ALT) levels greater than 10 times the upper limit of normal and elevated bilirubin levels.

4. Confirmation of Hepatitis A Virus Infection: The diagnosis of fulminant hepatitis A was confirmed by the detection of IgM-specific antibodies against hepatitis A virus using a validated diagnostic assay. This step ensured that patients included in the study had a confirmed diagnosis of hepatitis A.

**Exclusion criteria:** Patients with laboratory and pathological results favoring a diagnosis other than hepatitis A were excluded. Patients with a history of chronic liver disease or those presenting with markers associated with hepatitis B were also excluded.

**Data Collection:** The clinical and laboratory data of the enrolled patients were collected using specifically designed forms. Basic patient information, including age, sex, and history of primary liver disease, was recorded. Diagnostic markers, such as hepatitis A and B markers, as well as markers associated with autoimmune hepatitis, were assessed. Additionally, liver and coagulation tests were performed. The G6PD level was measured using a fluorescent spot test. Other laboratory parameters, including urine analysis, renal function criteria (BUN and Cr), and blood cell levels (WBC, Hb, PLT), were also measured. Information on the presence or absence of symptoms of encephalopathy, type of liver transplant performed (in case of transplantation), disease progression, need for transplantation, and mortality were recorded.

**Measurement of G6PD:** Glucose 6-phosphate dehydrogenase (G6PD) levels were measured using a fluorescent spot test. However, the fluorescent spot test is a commonly used method to assess G6PD activity. The fluorescent spot test provides a quantitative measurement of G6PD activity, allowing to determine the presence and severity of G6PD deficiency in individuals. The specific cut-off values or reference ranges used to categorize G6PD impairment are typically determined based on established clinical standards and guidelines.

**Statistical Analysis:** Statistical analysis was performed using SPSS Version 22. Descriptive statistics were presented as mean ± standard deviation for quantitative variables. Mann-Whitney test and Logistic Regression Overview were employed to investigate analytical findings between variables, while the Chi-square and t-tests were used for assessing descriptive data. A p-value of less than 0.05 was considered significant.

**Ethical Considerations:** The study was conducted in accordance with the ethical guidelines provided by the Shiraz University of Medical Ethics Committee. Informed consent was obtained from all participants, and patient confidentiality was maintained throughout the study. (IR.SUMS.MED.REC.1397.354).

## Results

A total of 81 patients with a mean age of 24 ± 4.6 (36-18) years were included in the present study, 57 (70.4%) patients were males, and 24 (29.6%) patients were females. G6PD deficiency was observed in 30 (37%) patients. None of the patients had a previous history of liver disease. Evidence of autoimmune hepatitis (positive for ANA anti-nucleotide antibody) was observed in 5 patients, but only in one case concurrence with autoimmune hepatitis was established. 

During treatment, 75 patients recovered at the end of the treatment period; one underwent liver transplant surgery, and five died. Table 1 shows the initial clinical information of patients. 

The laboratory results in table 2 indicate the presence of hepatitis in patients. An increase in the mean level of direct and total bilirubin and a decrease in the mean hemoglobin indicates the presence of hemolysis. Also, impairment in coagulation factors indicates the presence of liver failure in patients. 

**Table 1:** Preliminary clinical findings of patients

**Percentage**	**Number**	**variable**	
70.4	57	Male	**sex**
29.6	24	Female	
37	30	Yes	**G6PD Deficiency**
63	51	No	
43.2	35	Yes	**Encephalopathy**
56.8	46	No	
6.2	5	Positive	**ANA**
93.8	76	Negative	
75	75	Treated	**Disease outcome**
1.2	1	Liver transplanted	
6.2	5	Death	

G6PD.Glucose 6-phosphate dehydrogenase, ANA 2. Anti-Nuclear Antibodies

**Table 2:** Laboratory findings of patients at the time of admission

**Variable**	**Number**	**Minimum**	**Maximum**	**Mean**
**IGM HAV ** ^*^	81	2.00	22.00	10.63±3.42
**LDH**	79	214	19570	1400.68±2278.28
**RETIC**	76	0.05	21.00	3.24±3.72
**AST**	81	88	11350	2093.35±2220.49
**ALT**	81	118	15572	2879.31±2124.91
**ALK**	81	189	1066	429.68±177.82
**Alb**	81	2.4	4.4	3.44±0.42
**Glob**	81	2.1	7.0	3.67±0.85
**T bili**	81	3.1	84.0	27.15±19.17
**D bili**	81	1.7	37.0	14.36±8.89
**PT**	81	12.4	60.0	20.57±9.92
**PTT**	81	27	120	41.98±14.68
**INR**	81	1.00	14.00	2.51±2.27
**Hb **	81	6.0	16.5	11.52±2.65

*The normal value of the antibody level is less than 0.8. IGM HAV. Immunoglobulin M, Hepatitis A Virus, LDH. Lactate Dehydrogenase, RETIC. Reticulocyte, AST. Aspartate Aminotransferase, ALT. Alanine Aminotransferase, ALK. Alkaline Phosphatas, ALb. Albumin, Glob. Globulin, T bili. Total Bilirubin, D bili. Direct Bilirubin, PT. Prothrombin Time, PTT. Partial Thromboplastin Time, INR. International Normalized Ratio Hb. Hemoglobin

Table 3 shows the relationship between sex variables, the presence of encephalopathy, and the course of the disease in patients with and without G6PD deficiency. The results of the three tables show that the percentage of men with G6PD deficiency is significantly higher than women. The prevalence of this disorder is 42% in men with hepatitis fulminant A and 30% in women. The table above shows that 82.8% of patients with encephalopathy had G6PD dysfunction. The presence of encephalopathy was significantly higher in patients with G6PD deficiency than in patients without this disorder. Also, in examining the course of the disease in patients with and without G6PD deficiency, the results in the above table show that two of the patients died and the transplanted patient had G6PD deficiency, so the course of the disease in patients with G6PD deficiency. Significantly, it has a worse prognosis than patients without this defect.

**Table 3:** Relationship between sex variables, encephalopathy, and disease course in patients with and without G6PD deficiency

**Disease outcome**			**encephalopathy**		**sex**			
**Death**	**Liver transplant**	**Treated**	**No**	**Yes**	**female**	**male**		
5	1	24	1	29	6	24	**yes**	**G6PD deficiency**
0	0	51	45	6	18	33	**no**	
˂ 0.001			˂ 0.001		˂ 0.001		**p-value**	

Table 4 shows the laboratory parameters in patients with and without G6PD deficiency. As the results of the table above show, the levels of direct and total bilirubin, prothrombin time parameters (PT), PTT, and INR in patients with enzyme deficiency were significantly higher than in patients without this disorder. Also, the amount of hemoglobin in patients with G6PD deficiency was significantly lower than in patients without this disorder. 

Table 5 shows the laboratory parameters in patients with and without encephalopathy. As the results of the table above show, the levels of direct and total bilirubin, prothrombin time parameters (PT), PTT, and INR were significantly higher in patients with encephalopathy than in patients without this disorder. Also, the amount of hemoglobin and albumin in patients with encephalopathy was significantly lower than in patients without this disorder. 

**Table 4:** Laboratory parameters in patients with and without G6PD deficiency

**Variable**	**G6PD deficiency**	**Number**	**Mean±SD**	**P-value**
**ALT**	no	51	2979.88±2362.09	0.54
	yes	30	2708.33±1669.73	
**AST**	no	51	2067.69±2164.47	0.89
	yes	30	2136.97±2349.68	
**ALK**	no	51	417.35±165.93	0.41
	yes	30	450.63±197.58	
**Albumin**	no	51	3.50±0.46	0.09
	yes	30	3.35±0.35	
**globulin**	no	51	3.63±0.82	0.56
	yes	30	3.75±0.91	
**Total bilirubin**	no	51	16.62 ±11.7	0.00
	yes	30	45.06±15.86	
**Direct bilirubin**	no	51	9.95±6.33	0.00
	yes	30	21.86±7.53	
**PT**	no	51	18.63±6.95	0.021
	yes	30	23.88±3.05	
**PTT**	no	51	38.02±0.94	0.008
	yes	30	48.70±19.55	
**INR**	no	51	2.08±1.38	0.026
	yes	30	3.24±3.16	
**Hemoglobin**	no	51	12.93±1.99	0.00
	yes	30	9.13±1.76	

ALT. Alanine Aminotransferase, AST. Aspartate Aminotransferase, ALK. Alkaline Phosphatase, PT. Prothrombin Time, PTT. Partial Thromboplastin Time INR. International Normalized Ratio

**Table 5:** Laboratory parameters in patients with and without encephalopathy

**Variable**	**encephalopathy**	**Number**	**Mean±SD**	**P-value**
**ALT **	no	46	2713.91±1537.27	0.042
	yes	35	3096.69±2722.25	
**AST **	no	46	1908.04±1808.32	0.39
	yes	35	2336.89±2676.92	
**ALK **	no	46	422.80±169.55	0.69
	yes	35	438.71±190.2	
**PAlbumin ** ^4^	no	46	3.53±0.45	0.043
	yes	35	3.33±0.35	
**globulin**	no	46	3.58±0.82	0.28
	yes	35	3.79±0.90	
**Total bilirubin**	no	46	15.69±10.57	0.00
	yes	35	42.23±17.48	
**Direct bilirubin**	no	46	9.33±5.86	0.00
	yes	35	20.98±7.81	
**PT ** ^5^	no	46	17.48±6.53	0.001
	yes	35	24.63±12.06	
**PTT ** ^6^	no	46	36.76±7.27	0.00
	yes	35	48.83±18.7	
**INR ** ^7^	no	46	1.74±0.91	0.00
	yes	35	3.52±3.02	
**Hemoglobin**	no	46	12.99±1.92	0.00
	yes	35	9.60±2.22	
**IGM HAV ** ^8^	no	46	11.08±3.5	0.174
	yes	35	10.02±3.2	

ALT. Alanine Aminotransferase, AST. Aspartate Aminotransferase, ALK. Alkaline Phosphatase, PAlbumin. Plasma Albumin, PT. Prothrombin Time, PTT. Partial Thromboplastin Time, INR. International Normalized Ratio, IGM HAV. Immunoglobulin M, Hepatitis A Virus.

## Discussion

The present study aimed to investigate the clinical and laboratory characteristics of patients with hepatitis A virus (HAV) infection, with a specific focus on the presence of glucose-6-phosphate dehydrogenase (G6PD) deficiency and the occurrence of encephalopathy. The findings revealed several significant associations and shed light on the impact of these factors on patient outcomes. One of the key findings of this study was the higher prevalence of G6PD deficiency among male patients compared to females. The frequency of G6PD deficiency was observed to be 42% in males and 30% in females among those with fulminant hepatitis A. This difference in prevalence between genders highlights the potential role of genetic factors in modulating the susceptibility to G6PD deficiency in the context of HAV infection.

According to a research by Amir Aliabad et al., patients with hepatitis A were more likely to have G6PD deficiency than the general population at 37%. The prevalence of G6PD deficiency was reported to be 26% in children with hepatitis fulminant A (12), while a cross-sectional descriptive investigation on 1440 male subjects found that the prevalence of this condition was reported to be 7% in healthy subjects (14). 

12.5 % of the 280 kids in a Yasuj study showed a G6PD deficiency (18). In a meta-analysis study in Iran, the prevalence of G6PD deficiency in healthy persons was 6.7 percent .According to this study, the prevalence of this enzyme deficiency was 8.8% in men and 2.2% in women (19). Moreover, a notable association was identified between G6PD deficiency and the development of encephalopathy. The data demonstrated that a significant proportion (82.8%) of patients with encephalopathy exhibited G6PD dysfunction. This suggests that G6PD deficiency may predispose individuals to a higher risk of developing encephalopathy during HAV infection. The underlying mechanisms behind this association warrant further investigation. Sharma et al. found severe encephalopathy and multiple organ failure in their study's case report with a known G6PD deficiency and fulminant hepatitis A (13). 

The laboratory findings provided valuable insights into the disease pathophysiology and severity. Patients with G6PD deficiency exhibited elevated levels of direct and total bilirubin, indicating an impairment in bilirubin metabolism and clearance. Additionally, these patients displayed prolonged prothrombin time parameters (PT and PTT), increased international normalized ratio (INR), and decreased hemoglobin levels, suggesting impaired liver function and coagulation abnormalities.

 These laboratory abnormalities highlight the impact of G6PD deficiency on liver function and the potential for complications in patients with HAV infection. Furthermore, the presence of encephalopathy was associated with elevated levels of direct and total bilirubin, prolonged PT and PTT, increased INR, and decreased hemoglobin and albumin levels. These laboratory markers further support the severity of liver dysfunction and the association between encephalopathy and impaired liver function in HAV-infected patients.

Hepatitis and hemolysis can both generate elevated total bilirubin levels, which can result in misdiagnosis when hepatitis causes hemolysis in these individuals. Acute viral hepatitis, G6PD deficiency, and acute renal failure are all possible complications Contrary to adult patients. Children with G6PD deficiency seldom experience acute renal failure, unlike adult patient. However, acute viral hepatitis seldom has significant anemia (20). According to a research conducted in Nigeria (15), 24 % of individuals with acute viral hepatitis had anemia. The participants in the current research had an average hemoglobin level of 11.52 g/dl. In individuals with G6PD deficiency, this number was 9.13 g/dl; in those without this condition, it was 12.93 g/dl. It was statistically significant that there was a difference between the two groups (P= 0.001).

In the research by Amir Aliabad, there was no discernible difference in the hemoglobin level between children with fulminant hepatitis and those who did not have a G6PD deficit.

Overall, this study provides valuable insights into the clinical and laboratory characteristics of patients with HAV infection, particularly regarding the presence of G6PD deficiency and the occurrence of encephalopathy. Given the associations observed in this study, the evaluation of G6PD deficiency in patients with HAV infection emerges as a critical consideration in clinical practice.

The study was conducted at a single center, which may limit the generalizability of the findings. The retrospective nature of the study design may introduce biases and confounding factors. Additionally, the study's sample size and duration may impact the statistical power and generalizability of the results.

This study provides important insights into the clinical and laboratory characteristics of patients with hepatitis A virus infection, particularly regarding the presence of G6PD deficiency and the occurrence of encephalopathy. The findings demonstrate a significant association between G6PD deficiency and the development of encephalopathy, as well as their detrimental impact on patient outcomes. The identified laboratory parameters, including bilirubin levels, coagulation factors, and hemoglobin levels, serve as valuable indicators of liver function and disease severity in this patient population. These findings contribute to our understanding of the pathophysiology and clinical course of hepatitis A and underscore the importance of considering G6PD deficiency and encephalopathy in the management of these patients. Further research is warranted to explore potential therapeutic strategies targeting these specific risk factors to improve patient outcomes.
